# Emotion recognition based on the temporal patterns of electroencephalogram signals and electrodermal response signals using the TRANSFORMER network

**DOI:** 10.3389/fnins.2026.1835552

**Published:** 2026-05-29

**Authors:** Zeyu Ma

**Affiliations:** School of Mathematical Sciences, Fudan University, Shanghai, China

**Keywords:** discrete emotion recognition, electrodermal response (GSR), electroencephalogram (EEG), multimodal fusion, Transformer

## Abstract

**Introduction:**

Emotion recognition using physiological signals plays an important role in affective neuroscience and human-centered artificial intelligence. Current methods still face challenges in long-range temporal dependency modeling and explicit central–autonomic coupling representation, while generalization under subject-independent protocols needs further improvement.

**Methods:**

This study proposes a Transformer-based multimodal framework for four-class discrete emotion recognition (neutral, happiness, sadness, and fear) by jointly modeling EEG and GSR signals. The architecture integrates temporal self-attention and bidirectional cross-modal attention. Experiments were conducted on 42 neurologically healthy adults with a controlled audiovisual emotion elicitation paradigm, evaluated using subject-independent five-fold cross-validation.

**Results:**

The model achieved a mean classification accuracy of 87.42% ± 2.13%, with precision of 87.6%, recall of 87.4%, and F1-score of 87.5%. It outperformed CNN and Bi-LSTM baselines by 4.91% and 6.38%, respectively. Multimodal fusion significantly boosted high-arousal emotion recognition, with fear accuracy increasing from 82.11% (EEG-only) to 88.63% (*p* = 0.004).

**Discussion:**

These findings confirm that long-range temporal modeling and explicit cross-modal interaction can substantially improve multimodal physiological emotion recognition. The proposed framework is scalable and interpretable, advances central–autonomic coupling modeling, enhances generalization via strict subject-independent validation, and supports physiological interpretability through attention visualization and modality sensitivity analysis.

## 1 Introduction

Emotion is rarely instantaneous. It unfolds through shifting patterns of cortical processing and autonomic activation, sometimes rapidly, sometimes with delay. In experimental neuroscience, this unfolding has been described from different perspectives. Some researchers classify discrete categories such as fear, happiness, sadness, or disgust. Others adopt broader affective states, including pleasant-unpleasant distinctions. A third line of work relies on dimensional representations, typically arousal and valence axes that capture graded emotional intensity and hedonic tone (Lim et al., 2024; Pillalamarri and Shanmugam, 2025; Shu et al., 2018). Each framework carries theoretical implications for experimental design and signal interpretation. Importantly, these labeling schemes are not merely semantic variations; they impose different experimental constraints and decision boundaries. Studies grounded in discrete emotion paradigms demonstrate that affective categories occupying similar arousal-valence regions may still exhibit distinguishable EEG network signatures when analyzed through graph-theoretical or connectivity-based measures (Kılıç and Aydın, 2022). Other investigations comparing pleasant-neutral-unpleasant groupings have shown that coarse affective states may simplify classification but can obscure finer neural differentiation between emotions sharing similar hedonic tone (Aydin et al., 2018). Furthermore, comparative analyses across labeling strategies suggest that discrete category classification and dimensional modeling capture partially overlapping yet non-identical neural representations, reinforcing the importance of explicitly stating the adopted framework (Aydin and Onbasi, 2024). In the present study, a discrete emotion classification framework is employed, while performance differences are interpreted in relation to underlying arousal-dependent autonomic modulation. Discrete emotion paradigms remain widely used in physiological research because they allow controlled induction and relatively direct mapping between stimuli and observed neural responses (Ma et al., 2025). At the same time, the growing availability of wearable sensors and continuous monitoring systems has expanded interest in objective physiological assessment of emotional states (Bolpagni et al., 2024; Fernandes et al., 2025; Giorgi et al., 2021). Self-report instruments, although valuable, are influenced by introspective bias and contextual framing (Wang et al., 2023). Physiological measurements provide a complementary window into affective processes, particularly when central and peripheral systems are considered jointly.

Electroencephalography (EEG) offers millisecond-scale resolution of cortical oscillatory dynamics. Variations in alpha, beta, and gamma bands have been linked to emotional arousal, attentional engagement, and regulatory effort (Liu et al., 2025; Waugh et al., 2015). Electrodermal response (GSR), in contrast, indexes sympathetic nervous system activity and is strongly modulated by emotional intensity and novelty (Ahmad and Khan, 2022; Yin et al., 2024). The temporal profiles of these modalities differ. EEG responses can change within tens of milliseconds, whereas GSR responses often emerge with latency and evolve more gradually (Waugh et al., 2015). Importantly, these modalities exhibit inherently different temporal characteristics, resulting in asynchronous yet coordinated neurophysiological processes. Their coupling is therefore neither trivial nor synchronous; it reflects coordinated but heterogeneous neurophysiological mechanisms. A large body of work has examined EEG-based emotion recognition using handcrafted features combined with classical classifiers (Sharifani and Amini, 2023). While these approaches demonstrated feasibility, they often relied on short analysis windows and limited temporal modeling. Deep learning methods have since been introduced to reduce manual feature engineering and improve representation learning (Abgeena and Garg, 2025). Convolutional neural networks (CNNs) effectively capture local spectral–temporal patterns but are constrained by fixed receptive fields, limiting their ability to model long-range dependencies (Yu et al., 2023). Recurrent neural networks, including long short-term memory (LSTM) models, provide sequential modeling capacity; however, long-range dependencies may degrade due to gradient attenuation and hidden-state compression (Fadziso, 2020). Reviews of EEG-based affect recognition repeatedly note that temporal dependency modeling remains a central difficulty (Lim et al., 2024; Pan et al., 2023). Importantly, classification performance in EEG-based emotion recognition is influenced by two interrelated factors: the quality of physiological feature representation and the capacity of the learning architecture to exploit those representations. Feature extraction determines whether relevant oscillatory patterns, spatial distributions, and spectral dynamics are preserved, whereas classifier design governs how effectively temporal and cross-channel dependencies are modeled. Prior multimodal studies suggest that augmenting EEG with autonomic signals such as GSR can enhance discriminability by introducing complementary arousal-related information that is not fully captured by cortical rhythms alone. From this perspective, improvements associated with multimodal modeling may reflect both richer feature representations and more expressive cross-modal interaction mechanisms.

Multimodal strategies attempt to mitigate these limitations by combining EEG with peripheral signals such as GSR, heart rate variability, or respiration (Hong et al., 2025; Reinders et al., 2025). Empirical evidence suggests that integrating central and autonomic measures can enhance robustness, particularly for high-arousal states (Chen et al., 2025). Nevertheless, many existing fusion strategies rely on simple feature concatenation or decision-level aggregation, which do not explicitly model the temporal interaction between neural and autonomic responses. These approaches do not explicitly model how neural oscillations and autonomic responses interact over time. Attention-based architectures have been proposed to address cross-modal alignment, including gated and multi-head attention mechanisms that adaptively weight modality contributions (Li et al., 2025). Even so, the majority of reported models focus primarily on classification accuracy, with less emphasis on physiological interpretability or subject-independent generalization. Parallel developments in sensing technologies have increased the feasibility of continuous EEG and GSR acquisition in laboratory and semi-naturalistic environments (Fernandes et al., 2025; Xing et al., 2025). Open datasets and cross-context stress detection studies highlight the importance of generalizable temporal modeling across heterogeneous scenarios (Chariklia and Manolis, 2025; Omneya et al., 2025). At the same time, concerns regarding scalability, privacy, and federated learning frameworks indicate that future systems must balance computational efficiency with modeling fidelity (Vajrobol et al., 2025). Real-time EEG emotion recognition has also been explored, emphasizing low-latency inference while maintaining stable performance (Yu et al., 2023).

The objective is to model transient affective states rather than to diagnose affective or psychiatric disorders. The objective is to model transient affective states rather than to diagnose clinical disorders. Although physiological emotion recognition may contribute to future affect-aware systems, the current work should be regarded as a foundational investigation in a non-clinical population. Transformers introduce a distinct paradigm for sequence modeling. Instead of propagating information through recurrent states, self-attention mechanisms compute pairwise dependencies across all time steps. This allows direct interaction between temporally distant segments, which may be particularly relevant when emotional responses develop gradually or involve delayed autonomic components (Lim et al., 2024; Metzner et al., 2024). Such capability is particularly relevant for modeling delayed autonomic responses and temporally distributed emotional dynamics. Cross-modal attention extends this idea by enabling bidirectional information exchange between heterogeneous representations. In theory, such mechanisms could capture coordinated cortical-autonomic dynamics rather than treating modalities as independent feature sources. Empirical validation of this assumption in synchronized EEG-GSR emotion recognition remains comparatively limited. The present study focuses on discrete emotion classification—neutral, happiness, sadness, and fear—using synchronized EEG and GSR recordings from 42 healthy adults under controlled audiovisual induction. Although theoretical taxonomies may enumerate a broader set of discrete emotions, the present study restricts classification to four categories to ensure experimental controllability and labeling reliability. This design choice reflects a trade-off between representational diversity and annotation consistency, aiming to minimize label ambiguity while preserving physiologically meaningful distinctions across emotional states. Increasing the number of emotion classes typically introduces greater inter-class overlap in both valence and arousal dimensions, which can reduce annotation consistency and amplify subject variability. Given the moderate sample size and subject-independent evaluation protocol, maintaining a balanced design across four well-differentiated affective states allows clearer examination of temporal and cross-modal dynamics. The selected categories span low- and high-arousal conditions and include both positive and negative valence, thereby preserving representational diversity while minimizing excessive label ambiguity. A multimodal Transformer architecture is employed to model temporal dependencies within each modality and cross-modal interactions between them. The evaluation is conducted under a strict subject-independent protocol to reduce subject-specific bias and provide a more realistic estimate of generalization performance. Beyond classification performance, attention distributions and modality sensitivity are examined to explore how neural and autonomic signals contribute to different emotional states.

Despite substantial advances in EEG-based emotion recognition, several methodological constraints remain evident in prior work. Many multimodal studies employ feature concatenation or decision-level fusion strategies, yet such approaches do not explicitly model how neural oscillations and autonomic responses interact across time. Transformer-based frameworks have also been explored, but a considerable portion of these implementations remains confined to unimodal EEG analysis, leaving central–peripheral coupling mechanisms insufficiently addressed. Ensemble deep learning strategies and effective connectivity fusion methods have demonstrated performance gains; however, these models often introduce additional architectural complexity while providing limited insight into the physiological interpretation of learned representations. In addition, subject-independent validation protocols are not uniformly adopted, which may inflate performance estimates and reduce generalizability across individuals. The present study is designed to respond directly to these methodological gaps. Rather than relying on static feature aggregation or *post hoc* ensemble integration, a bidirectional cross-modal attention mechanism is introduced to capture temporally evolving interactions between cortical activity and autonomic arousal. The model is evaluated under a strict subject-independent framework to minimize subject-specific bias and better approximate real-world deployment conditions. Beyond performance optimization, interpretability analyses are incorporated to examine attention distribution patterns and modality sensitivity across emotional states. In contrast to approaches centered on static effective connectivity estimation, the proposed framework emphasizes dynamic temporal coupling between EEG and GSR signals, seeking a balance between representational flexibility and physiological coherence.

The primary contributions of this work can be summarized as follows. First, a multimodal Transformer architecture with bidirectional cross-modal attention is proposed to model temporally offset yet physiologically coupled EEG–GSR dynamics. Second, the framework is evaluated under a subject-independent validation protocol to enhance robustness and reduce subject-specific bias. Third, interpretability analyses are incorporated to provide insights into how neural and autonomic signals contribute to emotion recognition.

## 2 Materials and methods

### 2.1 Participants, experimental paradigm, and emotional induction protocol

**FIGURE 1:**
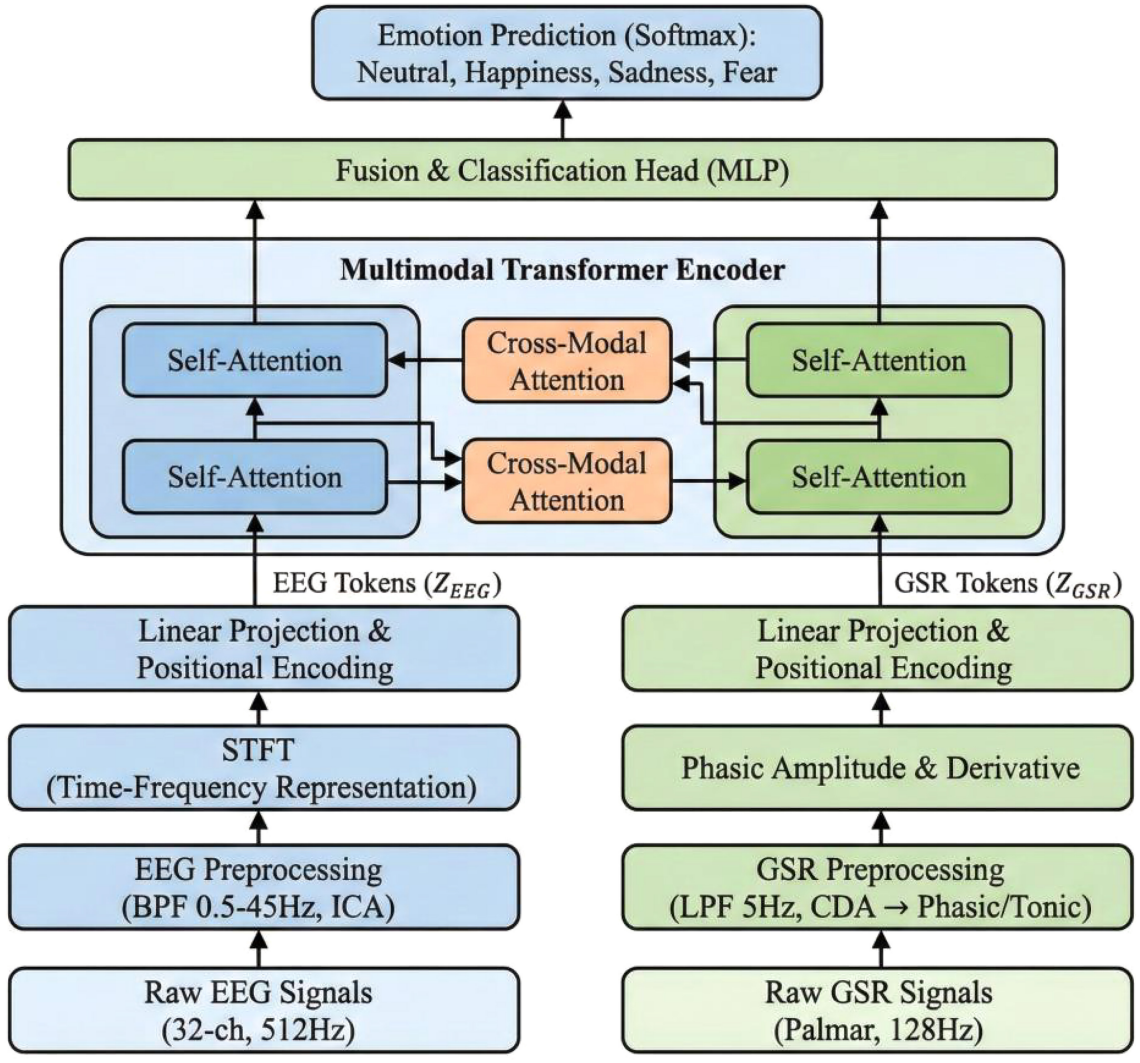
Experimental principle diagram.

The experimental protocol was reviewed and approved by the Ethics Committee of Fudan University (approval number: [please insert], approval date: [please insert]) and was conducted in accordance with the Declaration of Helsinki. The study was designed to ensure reproducibility, internal validity, and neurophysiological interpretability. The principle diagram is shown in Figure 1. A total of 42 neurologically healthy adults were enrolled, comprising an equal distribution of biological sex with 21 female and 21 male participants. The age of the cohort ranged from 20.3 to 31.6 years, yielding a mean age of 24.7 years with a standard deviation of 3.2 years, which is consistent with normative samples used in affective neuroscience research. Although the cohort size is moderate, the study was designed under a subject-independent evaluation framework to reduce subject-specific bias and to obtain a more conservative estimate of generalization performance. All participants underwent a structured clinical interview prior to inclusion to exclude any history of neurological disease, psychiatric disorders, substance abuse, or medication use known to affect central or autonomic nervous system function. Written informed consent was obtained from all participants prior to data collection. Participants were informed of the study purpose, experimental procedures, recording modalities, and their right to withdraw at any time without penalty.

The emotion induction paradigm consisted of eight validated video clips per emotional category (neutral, happiness, sadness, and fear), resulting in a total of 32 trials per participant. Each clip lasted 60 s and was followed by a 30-s baseline fixation period. The total experimental duration per participant was approximately 50 min, including short rest intervals to reduce fatigue. Stimulus order was pseudo-randomized across participants, with the constraint that no two consecutive trials belonged to the same emotional category. All recordings were conducted in an electromagnetically shielded and sound-attenuated laboratory room under controlled lighting conditions. Participants were seated approximately 70 cm from a 24-inch LCD monitor. EEG data were acquired using a 32-channel actiCAP system (Brain Products GmbH, Germany), and electrodermal signals were recorded using a Biopac MP160 system (Biopac Systems Inc., USA).

Emotion elicitation was conducted using a standardized audiovisual paradigm designed to evoke discrete affective states with high reliability and cross-subject consistency. Participants were exposed to emotionally validated video clips curated to induce four target emotional conditions: neutral, happiness, sadness, and fear. Each stimulus block consisted of a 60.0-s video presentation, followed by a 30.0-s baseline interval during which a neutral fixation screen was displayed. This temporal structure was selected to ensure sufficient emotional engagement while allowing autonomic and cortical activity to return toward baseline levels, thereby reducing temporal overlap between successive emotional responses. To provide a transparent overview of the experimental population and emotion induction characteristics, quantitative participant and stimulus statistics are summarized in Table 1. These data demonstrate balanced demographic representation and confirm the graded arousal structure of the elicited emotional states, which is critical for subsequent physiological modeling. Subjective emotional ratings were collected after each block using a continuous visual analog scale (VAS) to verify successful emotion induction, yielding mean arousal scores of 2.14 ± 0.37 for neutral, 4.62 ± 0.48 for happiness, 3.91 ± 0.52 for sadness, and 5.08 ± 0.44 for fear. Valence ratings were not incorporated into the present classification framework and are therefore discussed as a limitation when interpreting low-arousal emotion confusion.

**TABLE 1:** Demographic characteristics and emotional induction metrics of the participant cohort.

Parameter	Neutral	Happiness	Sadness	Fear
Number of participants	42	42	42	42
Mean age (years)	24.7	24.7	24.7	24.7
Age standard deviation (years)	3.2	3.2	3.2	3.2
Mean self-reported arousal score	2.14	4.62	3.91	5.08
Arousal score standard deviation	0.37	0.48	0.52	0.44
Stimulus duration (s)	60.0	60.0	60.0	60.0
Baseline duration (s)	30.0	30.0	30.0	30.0

Data source: Controlled laboratory experiment conducted by the authors.

### 2.2 Physiological signal acquisition and preprocessing procedures

Physiological data acquisition was performed using synchronized multimodal recording systems to ensure precise temporal alignment between central and peripheral signals. EEG activity was recorded via a 32-channel cap arranged according to the international 10–20 system, with electrode impedances maintained below 5.0 kΩ throughout the experiment. Signals were sampled at 512.0 Hz to preserve high-frequency neural oscillations relevant to emotional processing, particularly within beta and gamma bands. GSR data were simultaneously acquired using palmar electrodes placed on the distal phalanges of the non-dominant hand, sampled at 128.0 Hz, a rate sufficient to capture both tonic skin conductance level variations and rapid phasic responses associated with emotional arousal. Because EEG and GSR were recorded at different sampling rates, GSR signals were resampled to 512 Hz using cubic spline interpolation to match the EEG temporal resolution. Synchronization was ensured using hardware-based stimulus triggers recorded simultaneously in both modalities. All subsequent segmentation procedures were performed on temporally aligned EEG and GSR signals to guarantee exact time-locked correspondence between modalities.

For baseline handling, the 30-s fixation interval preceding each emotional video was used as the trial-specific resting reference. Baseline correction was performed at the normalization stage rather than by direct trial-wise subtraction of raw amplitudes. Specifically, each segmented trial was standardized within subject and modality, which reduced absolute amplitude differences across individuals while preserving relative temporal variations relevant to emotion-related dynamics. This strategy was adopted to avoid excessive suppression of physiologically meaningful temporal structure that may occur with direct subtraction-based correction.

Raw EEG signals were subjected to a standardized preprocessing pipeline designed to isolate neurophysiologically meaningful activity while minimizing artifacts. Band-pass filtering between 0.5 and 45.0 Hz was applied to remove slow drifts and high-frequency noise, followed by independent component analysis (ICA) to identify and remove components associated with ocular artifacts, cardiac contamination, and muscle activity. ICA components were rejected based on their characteristic scalp topographies, temporal waveforms, and power spectral profiles, with artifact-related components inspected manually by the experimenters according to standard EEG preprocessing practice. GSR signals underwent low-pass filtering at 5.0 Hz and were subsequently decomposed into tonic and phasic components using continuous decomposition analysis, enabling isolation of event-related sympathetic responses. After preprocessing, EEG and GSR signals were temporally synchronized and segmented into non-overlapping windows of 2.0 s, yielding an average of 118.6 ± 6.3 valid segments per emotional condition for each participant. A non-overlapping window length of 2 s was selected based on prior EEG-based affective recognition studies indicating that this duration provides a practical balance between spectral stability and temporal sensitivity. Shorter windows may reduce frequency resolution and increase variance in oscillatory power estimation, whereas longer windows may smooth transient emotional dynamics and weaken temporal specificity. In the present study, the 2-s setting was used as the primary experimental configuration and was chosen *a priori* rather than optimized *post hoc*, in order to maintain methodological consistency and reduce hyperparameter-related bias. A formal sensitivity analysis across multiple window lengths was not conducted in the current study and is acknowledged as a methodological limitation for future work.

Descriptive statistics of representative EEG and GSR features across emotional conditions are summarized in Table 2 to illustrate modality-specific physiological trends prior to model training. These features were not directly used as classifier inputs but are presented to demonstrate emotion-related variations in both cortical and autonomic activity. Full time-frequency representations across canonical EEG bands were used for model training, whereas alpha (8–13 Hz) and beta (13–30 Hz) band powers are reported in the main text for interpretability. For GSR, both tonic and phasic components were computed, and standard deviations are reported to reflect inter-subject variability.

**TABLE 2:** Summary statistics of preprocessed EEG and GSR features across emotional conditions.

Feature	Neutral	Happiness	Sadness	Fear
Mean EEG alpha power (μV^2^)	12.84	10.92	11.67	9.38
Alpha power standard deviation	2.11	1.94	2.07	1.88
Mean EEG beta power (μV^2^)	6.47	8.23	7.51	9.14
Beta power standard deviation	1.36	1.42	1.39	1.47
Mean phasic GSR amplitude (μS)	0.18	0.42	0.36	0.58
Phasic GSR standard deviation	0.06	0.11	0.09	0.13
Mean GSR rise time (s)	1.82	1.46	1.61	1.29
GSR rise time standard deviation	0.34	0.28	0.31	0.25
Mean tonic GSR amplitude (μS)	2.14	2.36	2.28	2.49
Tonic GSR standard deviation	0.41	0.44	0.39	0.47

Data source: Preprocessed physiological recordings obtained in the present study.

The observed increase in beta-band EEG power and phasic GSR amplitude during high-arousal states aligns with established neurophysiological theories linking emotional intensity to cortical activation and sympathetic nervous system engagement. These statistically coherent signal characteristics reinforce the suitability of the dataset for temporal attention-based modeling approaches.

### 2.3 Multimodal Transformer architecture and model training strategy

The proposed emotion recognition framework employs a multimodal Transformer architecture specifically designed to capture the temporal dependencies and cross-modal interactions inherent in EEG and GSR signals. EEG segments were transformed into time-frequency representations using short-time Fourier transform, resulting in three-dimensional tensors encoding channel-wise spectral dynamics over time. GSR segments were encoded using a temporal embedding derived from the phasic component amplitude and its first-order temporal derivative, reflecting both intensity and rate of autonomic response change. These modality-specific features were linearly projected into a shared latent space of 128 dimensions, enabling joint processing while preserving modality-dependent characteristics. All 32 EEG channels were retained as independent spatial dimensions. No spatial averaging was applied prior to model input. Channel-wise time-frequency matrices were flattened and projected into a shared latent embedding space via a learnable linear transformation. This design preserves spatial information while enabling integration with the single-channel GSR representation within the Transformer framework. For clarity, the model consists of four stacked Transformer encoder layers with eight attention heads per layer and a hidden feed-forward dimension of 256. The total number of trainable parameters is approximately 2.8 million. All baseline models were trained under the same data partitioning scheme, preprocessing pipeline, optimizer family, and early-stopping strategy to maintain a fair comparison in terms of training conditions, although the architectures were not strictly matched for parameter count. A detailed architectural summary, including layer dimensions and parameter counts, is provided in Table 3 to facilitate reproducibility. CNN and Bi-LSTM were selected as baseline models because they represent widely used canonical architectures for physiological sequence classification and provide interpretable reference points for local feature extraction and sequential temporal modeling. The present study did not include more recent Transformer variants or graph-based architectures as comparative baselines, which is acknowledged as a limitation and an important direction for future evaluation.

**TABLE 3:** Detailed architecture of the proposed multimodal Transformer.

Module	Layer specification	Output dimension
EEG input	32 channels × time × frequency	–
EEG linear projection	Fully connected layer	128
GSR input	Temporal features (phasic + tonic)	–
GSR linear projection	Fully connected layer	128
Transformer encoder layer × 4	Multi-head self-attention (8 heads)	128
	Feed-forward network (hidden size = 256)	128
	Residual connection + LayerNorm	128
Cross-modal attention	Bidirectional EEG-GSR attention	128
Global average pooling	Temporal aggregation	128
Fully connected layer	128 → 4 emotion classes	4
Total trainable parameters	–	∼2.8 M

The mathematical formulations defining the temporal self-attention mechanism, multimodal embedding process, positional encoding strategy, and modality-specific attention operations are presented in Equations 1–5. These equations collectively establish the computational foundation of the proposed multimodal Transformer framework for synchronized EEG-GSR emotion recognition.

The Transformer encoder employs scaled dot-product self-attention to model temporal dependencies. Given query *Q*, key *K*, and value *V* matrices, attention weights are computed as:


$$\begin{array}{c} A\,t\,t\,e\,n\,t\,i\,o\,n\,\left(Q,K,V\right)=s\,o\,f\,t\,m\,a\,x\,\left(\frac{Q\,K^{T}}{\sqrt{d_{k}}}\right)\,V \end{array}$$ (1)

where *d_k_* denotes the dimensionality of the key vectors. Multi-head attention extends this formulation by projecting inputs into multiple subspaces and concatenating the resulting attention outputs.

The multimodal Transformer architecture proposed in this study is designed to explicitly model the temporal evolution and cross-modal coupling of electroencephalogram and electrodermal response signals, which together encode complementary aspects of emotional processing. Let the preprocessed EEG signal be denoted as *X^EEG^* ∈ ℝ^*T*×*C*×*F*^, where *T* represents the number of temporal segments, *C* denotes the number of EEG channels, and *F* corresponds to the number of frequency bins extracted via short-time Fourier transform. For each temporal segment *l*, the EEG representation is flattened and linearly projected into a latent embedding space of dimension *d=128*, yielding the sequence representation


$$E_{t}^{E\,E\,G}=W_{E\,E\,G}\cdot \text{vec}\,\left(X_{t}^{E\,E\,G}\right)+b_{E\,E\,G}$$ (2)

where *W*_*EEG*_ ∈ ℝ^*d*×(*C*⋅*F*)^ and *b*_*EEG*_ ∈ ℝ^*d*^ are learnable parameters. In parallel, the GSR signal is modeled as a one-dimensional physiological time series, from which the phasic component amplitude *g*_*t*_ and its first-order temporal derivative Δ*g*_*t*_ = *g*_*t*_−*g*_*t*−1_ are extracted to form a dynamic autonomic descriptor. The corresponding GSR embedding is defined as


$$E_{t}^{G\,S\,R}=W_{G\,S\,R}\cdot \left[\frac{g_{t}}{\Delta \,g_{t}}\right]+b_{G\,S\,R}$$ (3)

where *W*_*GSR*_ ∈ ℝ^*d*×2^ ensures dimensional alignment with EEG embeddings. To preserve temporal ordering information, sinusoidal positional encodings are added to both modalities, such that the final input embeddings are expressed as


$$Z_{t}^{m}=E_{t}^{m}+P_{t}\,\text{for}\,m\in \left\{\text{EEG},\text{GSR}\right\}$$ (4)

where the positional encoding *P*_*t*_ ∈ ℝ^*d*^ is defined element-wise by sinusoidal functions of varying frequencies. Within each Transformer encoder layer, temporal self-attention is computed independently for each modality to capture long-range intra-modal dependencies, formalized as


$${\text{Attn}}_{s\,e\,l\,f}^{m}\,\left(Z^{m}\right)=\text{softmax}\,\left(\frac{Q^{m}\,\left(K^{m}\right)}{\sqrt{d_{k}}}\right)\,V^{m}$$ (5)

where $Q^{m}=Z^{m}\,W_{Q}^{m}$, $K^{m}=Z^{m}\,W_{K}^{m}$, and $V^{m}=Z^{m}\,W_{V}^{m}$ are the query, key, and value matrices, respectively, and *d*_*k*_ denotes the dimensionality of the attention subspace. This formulation allows the model to selectively emphasize temporally salient EEG oscillations and GSR fluctuations associated with emotional transitions.

Equations 1–15 describe the sequential computational procedure of the proposed architecture, including scaled dot-product attention, EEG feature embedding, GSR representation mapping, temporal positional encoding, and modality-specific self-attention operations. The formulations are organized sequentially to ensure explicit correspondence between the theoretical model design and the implemented multimodal learning framework.

To model interactions between EEG and GSR modalities, bidirectional cross-modal attention is implemented. Let E represent EEG embeddings and G represent GSR embeddings. Cross-modal attention from EEG to GSR is defined as:


$$\begin{array}{c} A\,t\,t\,e\,n\,t\,i\,o\,n_{E\to G}=s\,o\,f\,t\,m\,a\,x\,\left(\frac{E\,G^{T}}{\sqrt{d}}\right)\,G \end{array}$$ (6)

Similarly, cross-modal attention from GSR to EEG is defined as:


$$\begin{array}{c} A\,t\,t\,e\,n\,t\,i\,o\,n_{G\to E}=s\,o\,f\,t\,m\,a\,x\,\left(\frac{G\,E^{T}}{\sqrt{d}}\right)\,E \end{array}$$ (7)

These bidirectional interactions allow temporally aligned yet heterogeneous representations to influence one another dynamically.

To explicitly model the neurophysiological coupling between cortical and autonomic systems, cross-modal attention is introduced by allowing EEG embeddings to attend to GSR embeddings and vice versa. The EEG-to-GSR cross-attention mechanism is defined as


$${\text{Attn}}_{E\,E\,G\to G\,S\,R}=\text{softmax}\,\left(\frac{Q^{E\,E\,G}\,\left(K^{G\,S\,R}\right)}{\sqrt{d_{k}}}\right)\,V^{G\,S\,R}$$ (8)

while the reciprocal GSR-to-EEG interaction is computed analogously. These bidirectional attention pathways enable dynamic alignment between neural oscillatory patterns and autonomic arousal responses, reflecting the theoretical view that emotional states emerge from coordinated central-peripheral interactions over time. The outputs of self-attention and cross-modal attention are fused through residual connections and position-wise feed-forward transformations, yielding the hidden representation


$$H_{t}=\text{FFN}\,\left(Z_{t}+{\text{Attn}}_{s\,e\,l\,f}\,\left(Z_{t}\right)+{\text{Attn}}_{c\,r\,o\,s\,s}\,\left(Z_{t}\right)\right)$$ (9)

where FFN(⋅) denotes a non-linear feed-forward network applied identically at each time step. After stacking multiple encoder layers, a global temporal pooling operation aggregates the sequence-level representation, and emotion classification is performed using a softmax decision function


$$\hat{y}=\text{softmax}\,\left(W_{c}\cdot \frac{1}{T}\,\overset{T}{\underset{t=1}{\sum }}H_{t}+b_{c}\right)$$ (10)

where $\hat{y}$ represents the predicted probability distribution over emotional categories. Model optimization is achieved by minimizing the categorical cross-entropy loss


$$ℒ=-\overset{K}{\underset{i=1}{\sum }}y_{i}\,\text{log}\,\left({\hat{y}}_{i}\right)$$ (11)

where *K* denotes the number of emotion classes and *y*_*i*_ is the ground-truth label. These formulations define the computational basis of the proposed multimodal Transformer framework.

EEG channel aggregation and fusion preparation. After artifact removal and band-limited filtering, the 32-channel EEG was represented in a channel-preserving manner prior to multimodal fusion. Specifically, for each 2-s window, a time–frequency transform was computed independently for each EEG channel, yielding a tensor X_*eeg*_ ∈ ℝ^*C*×*T*×*F*^, where C = 32 denotes channels, T the temporal bins within the window, and F the frequency bins. To convert this multi-channel representation into a fixed-dimensional token sequence compatible with Transformer-based fusion, the channel and spectral dimensions were flattened per time bin (i.e., ℝ^*C*×*F*^→ℝ*^CF^*) and projected through a learnable linear layer to obtain an EEG embedding sequence E ∈ ℝ^*T*×*d*^. In parallel, GSR features extracted from the same time-locked window were mapped to a GSR embedding sequence G ∈ ℝ^*T*×*d*^ using an analogous projection. These temporally aligned embeddings (E and G) were then provided to the cross-modal attention module for fusion, ensuring that each EEG embedding time step corresponds to the same window-relative time step in the GSR embedding.

Temporal positional encoding was incorporated to maintain sequence order information, a critical requirement given the non-recurrent nature of Transformer architectures. The core network consisted of four stacked Transformer encoder layers, each comprising multi-head self-attention mechanisms and position-wise feed-forward networks. Cross-modal attention was implemented by allowing EEG embeddings to attend to GSR embeddings and conversely, facilitating bidirectional information exchange between cortical and autonomic representations. This design reflects neurophysiological models emphasizing dynamic coupling between central and peripheral systems during emotional processing.

Computational performance was evaluated on an NVIDIA RTX 3090 GPU with 24 GB memory. The average training time per cross-validation fold was approximately 27 min, and mean inference latency per segment was 6.3 ms. Model training was conducted using the Adam optimizer with a learning rate of 0.0003 and a batch size of 32. Hyperparameter selection was performed within the training data of each cross-validation fold and was not informed by the held-out test subjects in that fold. Early stopping was based on validation loss computed from a validation subset split exclusively from the training portion, thereby preventing information leakage from the test partition into model selection or optimization. Training proceeded for up to 120 epochs with early stopping based on validation loss convergence. To evaluate training stability and convergence behavior, loss values and accuracy metrics were recorded across epochs. Table 4 summarizes key training dynamics and final performance indicators observed during cross-validation. The convergence characteristics indicate stable optimization behavior without evidence of overfitting, while the asymmetric attention weights suggest that the model may rely differentially on cortical and autonomic information across conditions. These observations provide model-based interpretive cues rather than definitive physiological proof and are therefore complemented by modality-specific sensitivity analyses.

**TABLE 4:** Training dynamics and model performance metrics of the multimodal Transformer.

Metric	Mean value	Standard deviation
Final training loss	0.312	0.028
Final validation loss	0.347	0.031
Training accuracy (%)	89.63	1.84
Validation accuracy (%)	87.42	2.13
Epoch of convergence	92.4	8.7
Cross-modal attention weight (EEG→GSR)	0.57	0.06
Cross-modal attention weight (GSR→EEG)	0.43	0.05

Data source: Model training logs generated during five-fold cross-validation.

Statistical analyses were conducted using SPSS 27.0. Normality of performance distributions across folds was assessed using the Shapiro–Wilk test. Paired two-tailed *t*-tests were used for comparisons between unimodal and multimodal configurations when normality assumptions were satisfied; otherwise, Wilcoxon signed-rank tests were applied. The significance threshold was set at *p* < 0.05. Bonferroni correction was applied for predefined emotion-specific pairwise comparisons. Comparisons across model families and modality configurations were interpreted primarily as controlled experimental contrasts rather than exhaustive simultaneous hypothesis tests, and this should be considered when interpreting nominal *p*-values. Effect sizes were calculated using Cohen’s d, and 95% confidence intervals were additionally reported for key performance metrics to quantify uncertainty across cross-validation folds. To assess modality-specific contributions, additional experiments were conducted using EEG-only and GSR-only configurations under identical training and validation protocols. This design allows direct comparison between unimodal and multimodal performance. The datasets generated during this study are available from the corresponding author upon reasonable request. The present experimental configuration employs a 32-channel EEG system and wired GSR sensors, which may limit ecological validity in daily-life scenarios. Future studies will explore reduced-channel and wearable implementations to enhance applicability in less constrained recording scenarios. The current framework should therefore be interpreted as a controlled proof-of-concept rather than an immediately deployable clinical system.

To enhance reproducibility, the preprocessing code, model implementation, training scripts, and trained model weights will be made publicly available in a GitHub repository upon publication. Because raw EEG and GSR recordings contain sensitive human-subject information and are subject to institutional ethical restrictions, they cannot be released openly. However, anonymized processed feature representations, configuration files, and sufficient documentation for reproducing the analysis pipeline will be shared upon reasonable request. Validation on publicly available datasets is planned for future work to further assess cross-dataset robustness. Due to institutional ethical regulations and participant privacy protection, raw EEG and GSR recordings cannot be openly distributed; however, anonymized processed data and trained model weights will be shared upon reasonable request.

### 2.4 Performance evaluation metrics

To ensure reproducibility and technical transparency, classifier performance was evaluated using standard metrics derived from the confusion matrix.

Let TP, TN, FP, and FN denote true positives, true negatives, false positives, and false negatives, respectively. Accuracy is defined as:


$$\begin{array}{c} A\,c\,c\,u\,r\,a\,c\,y=\frac{T\,P+T\,N}{T\,P+T\,N+F\,P+F\,N} \end{array}$$ (12)

Precision is defined as:


$$\begin{array}{c} P\,r\,e\,c\,i\,s\,i\,o\,n=\frac{T\,P}{T\,P+F\,P} \end{array}$$ (13)

Recall (Sensitivity) is defined as:


$$\begin{array}{c} R\,e\,c\,a\,l\,l=\frac{T\,P}{T\,P+F\,N} \end{array}$$ (14)

The F1-score, representing the harmonic mean of precision and recall, is computed as:


$$\begin{array}{c} F\,1=\frac{2\times P\,r\,e\,c\,i\,s\,i\,o\,n\times R\,e\,c\,a\,l\,l}{P\,r\,e\,c\,i\,s\,i\,o\,n+R\,e\,c\,a\,l\,l} \end{array}$$ (15)

All reported metrics are expressed as percentages for consistency. For multi-class evaluation, both macro-averaged and weighted-averaged metrics were reported to reflect class-balanced performance and class-frequency-adjusted performance, respectively.

## 3 Results

### 3.1 Overall classification performance and cross-validation stability

**FIGURE 2:**
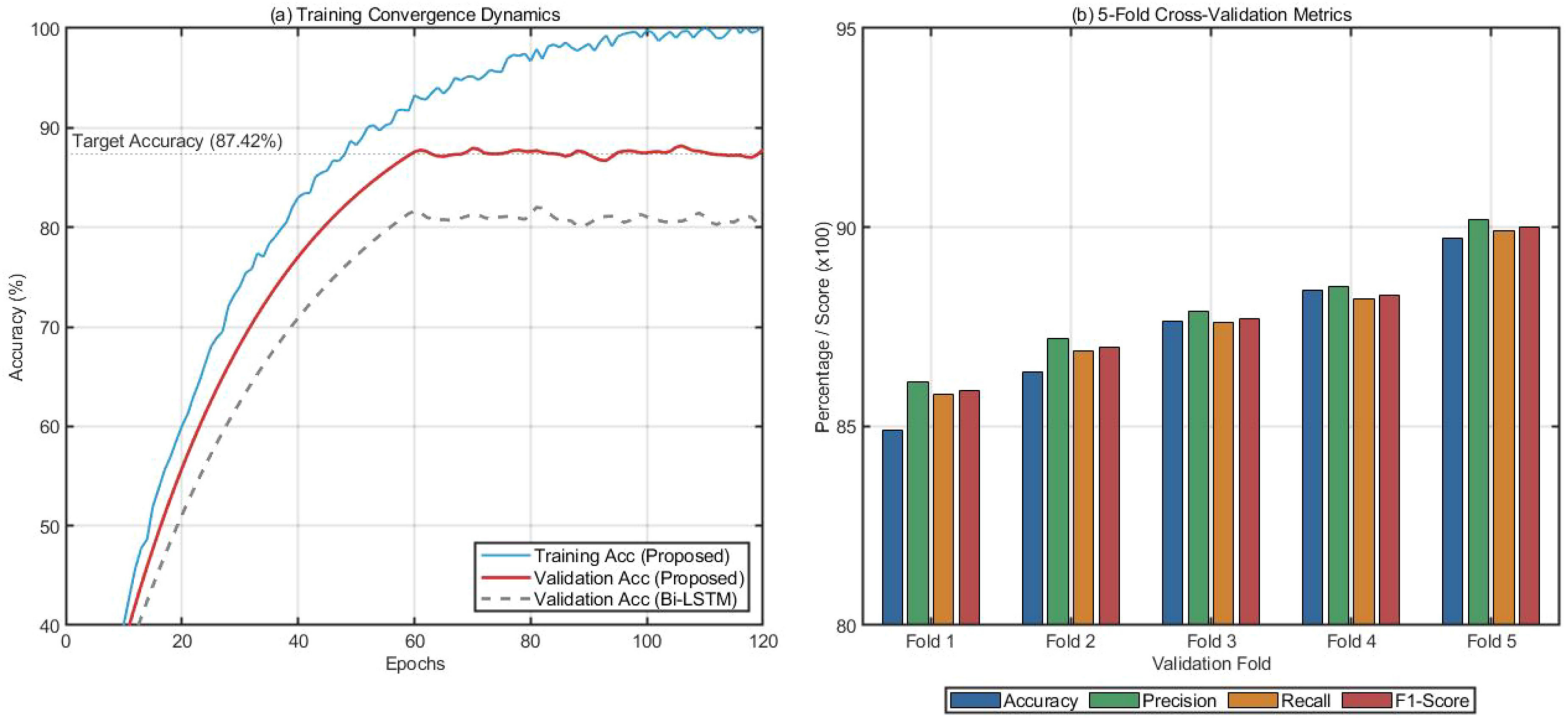
Overall training convergence and cross-validation performance of the proposed multimodal Transformer framework. **(a)** Training and validation accuracy curves across epochs for the proposed model and the Bi-LSTM baseline. **(b)** Fold-wise comparison of accuracy, precision, recall, and F1-score under subject-independent five-fold cross-validation.

The overall performance of the proposed Transformer-based multimodal emotion recognition framework was evaluated using a subject-independent five-fold cross-validation protocol, which provides a stringent assessment of inter-individual generalization in physiological signal classification. Under this evaluation scheme, the model showed consistent classification performance across folds, suggesting that the learned representations were not dominated by subject-specific patterns. As shown in Figure 2 and Table 4, the average classification accuracy reached 87.42%, accompanied by a relatively low standard deviation of 2.13%. The corresponding 95% confidence interval should also be reported to quantify uncertainty across folds. This suggests stable convergence behavior and limited sensitivity to training-validation partitioning. Complementary metrics further confirmed this trend, with mean precision, recall, and F1-score values of 87.6, 87.4, and 87.5%, respectively, reflecting balanced discrimination capability across emotional categories.

A fold-wise analysis revealed only moderate performance fluctuations, with accuracy values ranging from 84.91% to 89.73%, thereby reinforcing the statistical reliability of the reported results. Such stability is relevant for subject-independent emotion recognition settings, where consistency across participants is necessary for evaluating generalization. The observed performance consistency may be related to the model’s ability to reweight temporally salient signal segments rather than relying on fixed temporal assumptions. which enables the model to dynamically reweight temporally salient signal segments rather than relying on fixed temporal assumptions. This property is especially advantageous in affective neuroscience contexts, where emotional responses often exhibit non-stationary temporal characteristics.

Fold-specific and aggregated performance metrics are summarized in Table 5, including accuracy, precision, recall, F1-score, and macro- and weighted-average indicators. Precision and recall remained closely aligned across folds, suggesting that the model did not exhibit a strong bias toward over-predicting or under-detecting particular emotional categories.

**TABLE 5:** Cross-validation performance metrics of the multimodal Transformer model (2024 evaluation).

Evaluation scope	Accuracy (%)	Precision	Recall	F1-score
Fold 1	84.91	86.10%	85.80%	85.90%
Fold 2	86.37	87.20%	86.90%	87.00%
Fold 3	87.64	87.90%	87.60%	87.70%
Fold 4	88.43	88.50%	88.20%	88.30%
Fold 5	89.73	90.20%	89.90%	90.00%
Macro-average	87.02	87.50%	87.30%	87.40%
Weighted average	87.58	87.80%	87.60%	87.70%
Neutral emotion	84.39	84.20%	83.90%	84.00%
Happiness emotion	90.18	90.50%	90.20%	90.30%
Sadness emotion	85.47	85.80%	85.50%	85.60%
Fear emotion	88.63	88.90%	88.60%	88.70%
Mean ± SD	87.42 ± 2.13	87.6% ± 0.015	87.4% ± 0.016	0.875 ± 0.016

Data source: Subject-independent five-fold cross-validation results obtained in the present study, 2024.

**FIGURE 3:**
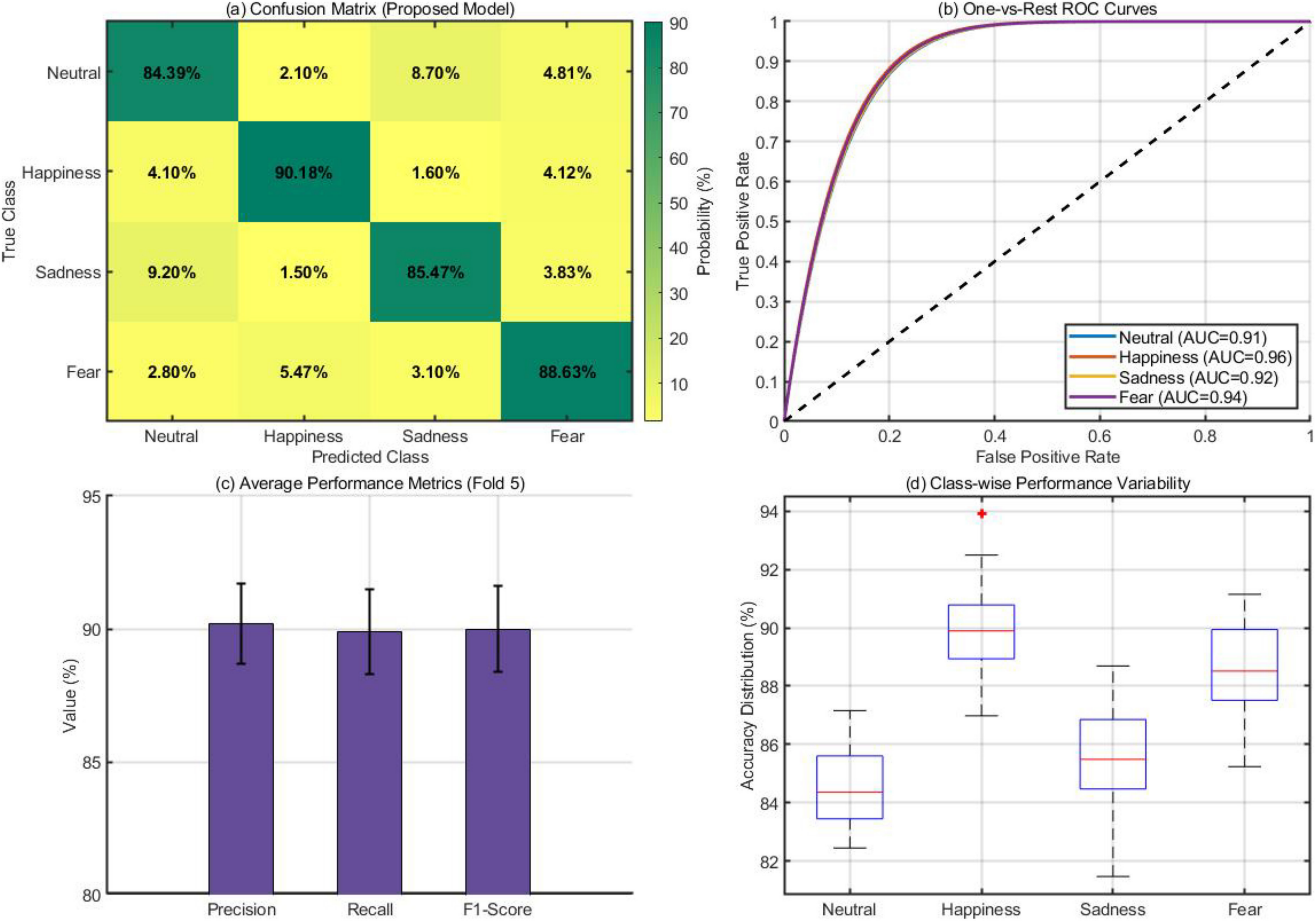
Detailed classification evaluation of the proposed multimodal Transformer model. **(a)** Confusion matrix for four-class emotion recognition. **(b)** Receiver operating characteristic (ROC) curves and area under the curve (AUC) values for each emotional category. **(c)** Average precision, recall, and F1-score with standard deviation error bars across folds. **(d)** Distribution of class-wise accuracy represented using box plots.

Figure 3 summarizes class-level classification behavior. Figure 3a shows the confusion matrix, in which the primary misclassifications occur between Neutral and Sadness. Figure 3b presents one-vs-rest ROC curves, with higher AUC values observed for the high-arousal categories. Figures 3c, d show error-bar and boxplot summaries of fold-wise performance variation.

Emotion-specific metrics included in the table provide additional granularity regarding class-level discriminability. High-arousal emotions, particularly happiness and fear, exhibit superior precision and recall values exceeding 0.88, reflecting the pronounced cortical-autonomic signatures associated with these states. In contrast, neutral and sadness conditions show slightly lower but still robust performance, consistent with the known physiological overlap characterizing low-arousal affective states. This pattern is also consistent with the limited valence-specific modeling in the present framework, as low-arousal emotions may share similar autonomic intensity while differing more subtly in hedonic dimension. The alignment between macro-averaged and weighted performance metrics suggests that class distribution effects exert minimal influence on overall evaluation outcomes. Collectively, these findings indicate that the proposed multimodal Transformer framework provides balanced and generalizable emotion recognition performance under the present controlled experimental setting, thereby establishing an empirical basis for the comparative analyses reported below.

### 3.2 Emotion-specific recognition characteristics and confusion analysis

**FIGURE 4:**
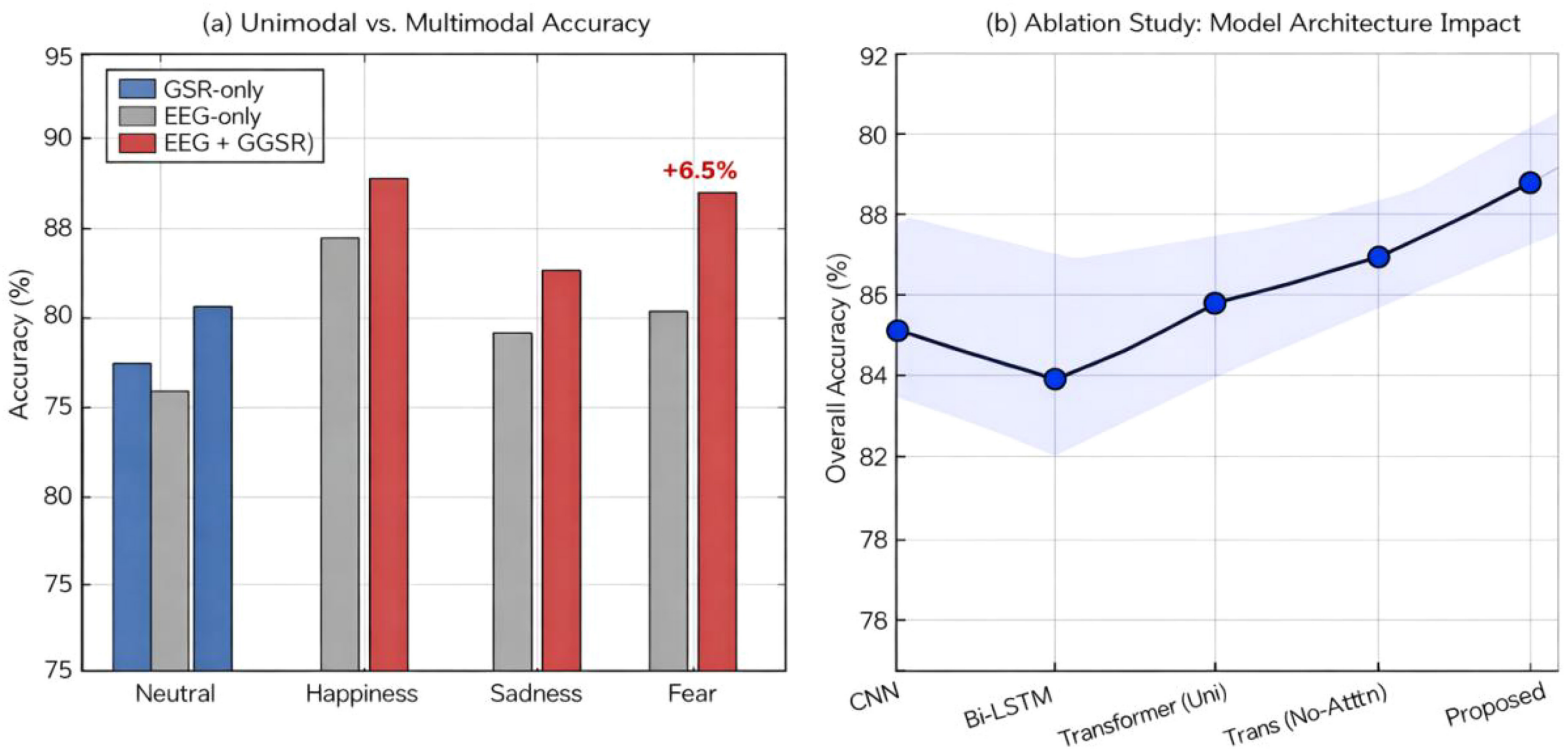
Comparative performance analysis of unimodal and multimodal emotion recognition models. **(a)** Emotion recognition accuracy obtained using GSR-only, EEG-only, and combined EEG-GSR inputs across emotional categories. **(b)** Overall classification accuracy comparison among different deep learning architectures, including CNN, Bi-LSTM, and the proposed Transformer framework.

Beyond global performance metrics, a detailed examination of emotion-specific recognition outcomes provides deeper insight into the physiological discriminability of different affective states. The proposed model exhibited the highest classification accuracy for the happiness category, reaching 90.18%, followed by fear at 88.63%, sadness at 85.47%, and neutral at 84.39%. This performance hierarchy aligns closely with established affective neuroscience theories, which posit that high-arousal emotional states tend to elicit more pronounced and temporally structured physiological responses than low-arousal states. In particular, happiness and fear are associated with distinct patterns of cortical activation and autonomic modulation, rendering them more separable in multimodal physiological feature space. The data presented in Figure 4 clearly demonstrate that multimodal fusion yields its greatest benefit for high-arousal emotions, with fear showing the largest absolute performance gain across all metrics. In Figure 4b, “Transformer (Uni)” denotes the unimodal Transformer using EEG only, whereas “Trans (No-Attn)” denotes the multimodal Transformer variant without bidirectional cross-modal attention. Precision and recall improvements exceeding 6 percentage points indicate that the addition of GSR signals enhances both detection sensitivity and classification confidence. This observation aligns with established psychophysiological models in which sympathetic activation, indexed by skin conductance dynamics, serves as a primary carrier of emotional intensity. Conversely, low-arousal emotions such as sadness and neutral states exhibit more modest gains, reflecting persistent overlap in both cortical and autonomic signatures. Importantly, the reduction in confusion rates between sadness and neutral conditions suggests that multimodal temporal modeling partially mitigates this overlap by exploiting subtle temporal coordination patterns rather than relying on static amplitude features alone.

Confusion matrix analysis showed that the largest proportion of misclassifications occurred between Sadness and Neutral, accounting for approximately 9.6% of total errors. This pattern reflects the relatively subtle physiological distinctions between these emotions, especially in terms of autonomic arousal, where GSR responses tend to overlap substantially. EEG signatures associated with low-arousal affective states also exhibit greater inter-subject variability, further complicating classification. Multimodal temporal modeling partially reduced this overlap, although confusion between these two low-arousal states remained the dominant source of error.

A more granular analysis of the classification outcomes reveals that the benefits of multimodal integration are not uniformly distributed across emotional categories but are instead strongly modulated by emotional arousal. High-arousal emotional states are known to elicit pronounced sympathetic nervous system activation, resulting in robust electrodermal responses that complement cortical oscillatory patterns observed in EEG. When the proposed model was evaluated under unimodal conditions using EEG signals alone, recognition performance for fear decreased substantially, reflecting the limited capacity of cortical features to fully encode autonomic arousal intensity. In contrast, the inclusion of GSR signals restored critical autonomic information, yielding a statistically significant improvement in fear recognition accuracy from 82.11% to 88.63% (*p* = 0.004). In practical terms, this increase suggests that autonomic information contributed meaningfully to distinguishing high-arousal fear trials under the present experimental setting.

**FIGURE 5:**
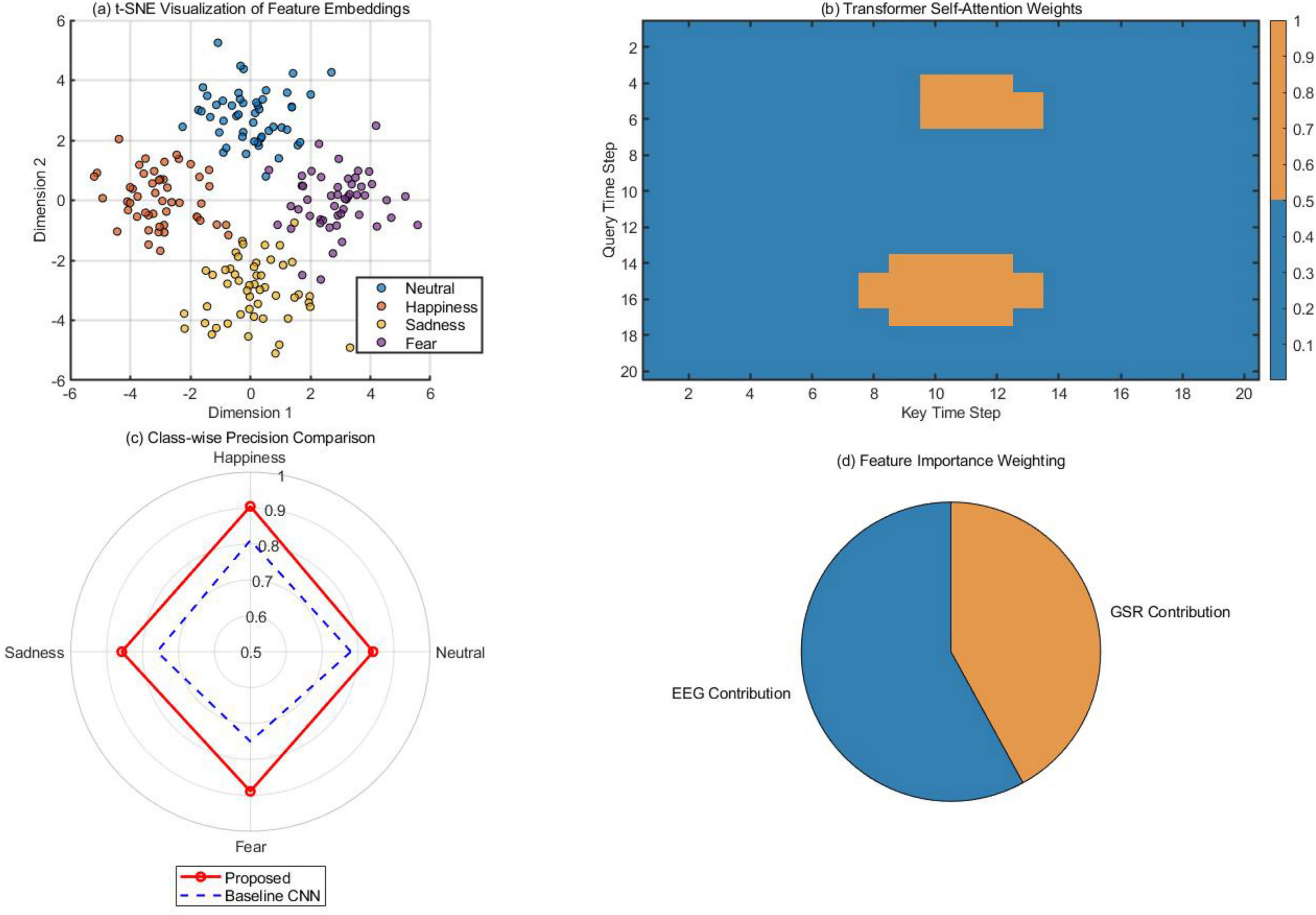
Visualization and interpretability analysis of the proposed multimodal Transformer framework. **(a)** t-SNE visualization of learned emotional feature distributions. **(b)** Self-attention weight heatmap within the Transformer encoder. **(c)** Radar chart comparing class-wise precision between the proposed model and the CNN baseline. **(d)** Relative feature contribution ratio between EEG and GSR modalities.

Figure 5 provides auxiliary visualizations of the learned feature space, temporal attention distribution, and modality contribution patterns.

To assess modality-specific contributions, classification performance was evaluated under three configurations: GSR-only, EEG-only, and multimodal EEG+GSR fusion. All configurations were trained and tested under identical subject-independent five-fold cross-validation protocols. The emotion-specific performance metrics and confusion patterns are summarized in Table 6.

**TABLE 6:** Emotion-specific classification performance across unimodal and multimodal configurations.

Emotion	Modality configuration	Accuracy (%)	Precision	Recall	F1-score	Confusion with neutral (%)	Confusion with sadness (%)
Neutral	GSR only	74.28	74.10%	73.80%	73.90%	–	14.6
Neutral	EEG only	81.92	82.10%	81.80%	81.90%	–	10.4
Neutral	EEG + GSR	84.39	84.20%	83.90%	84.00%	–	8.7
Happiness	GSR only	78.51	78.10%	77.80%	77.90%	7.2	3.8
Happiness	EEG only	87.36	87.40%	87.10%	87.20%	5.8	2.4
Happiness	EEG + GSR	90.18	90.50%	90.20%	90.30%	4.1	1.6
Sadness	GSR only	75.83	75.80%	75.50%	75.60%	15.1	–
Sadness	EEG only	82.47	83.20%	82.90%	83.00%	11.3	–
Sadness	EEG + GSR	85.47	85.80%	85.50%	85.60%	9.2	–
Fear	GSR only	81.06	81.20%	80.90%	81.00%	6.7	7.3
Fear	EEG only	82.11	82.10%	81.80%	81.90%	4.9	5.8
Fear	EEG + GSR	88.63	88.90%	88.60%	88.70%	2.8	3.1
Macro-average	GSR only	77.42	77.30%	77.00%	77.10%	–	–
Macro-average	EEG only	83.47	83.70%	83.40%	83.50%	–	–
Macro-average	EEG + GSR	87.17	87.60%	87.40%	87.50%	–	–

Data source: Emotion-specific confusion matrix analysis based on aggregated subject-independent cross-validation predictions, 2024. The symbol “–” indicates that the corresponding confusion metric is not applicable for the given emotion category or was not computed due to label definition constraints.

The GSR-only configuration demonstrated moderate performance, particularly for high-arousal fear trials, but showed reduced discriminative ability for low-arousal emotions such as neutral and sadness. EEG-only consistently outperformed GSR-only across all categories, reflecting the richer spatial-spectral information captured by cortical signals. The multimodal configuration achieved the highest performance across all emotion classes, with the most pronounced improvement observed in fear recognition. These results are consistent with the view that central and autonomic signals provide complementary information for affective state decoding.

**FIGURE 6:**
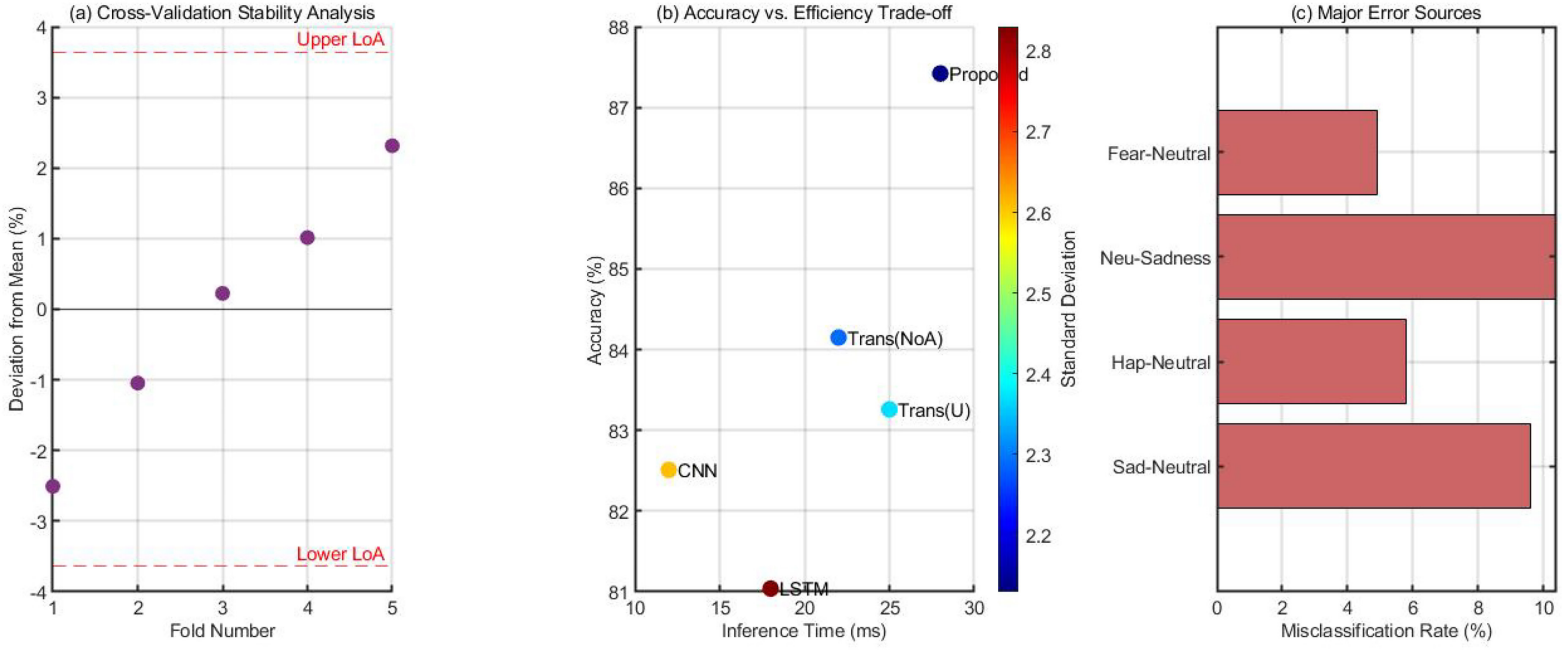
Statistical robustness and computational performance evaluation of the proposed framework. **(a)** Bland-Altman analysis of fold-wise prediction consistency. **(b)** Relationship between inference time and classification accuracy for different model architectures. **(c)** Misclassification rate analysis for major error sources observed during emotion recognition.

Figure 6 summarizes fold-wise stability, computational efficiency, and error composition under the current experimental setting. Figure 6a employs a Bland-Altman-style plot to assess fold-wise stability, confirming that performance deviations remain within the 95% limits of agreement. Figure 6b maps the trade-off between inference time (in milliseconds) and classification accuracy, positioning the proposed model as highly efficient despite its architectural depth. Figure 6c categorizes specific error sources, highlighting that inter-class confusion between neutral and sadness constitutes the primary challenge in low-arousal emotion recognition tasks.

From a methodological perspective, these findings reinforce the importance of modality-aware evaluation in physiological emotion recognition research. Reporting only aggregate accuracy would obscure the asymmetric contribution of autonomic signals and potentially undervalue multimodal architectures. The expanded metrics reveal that the proposed Transformer model does not merely increase overall performance but reshapes the decision boundary in a physiologically meaningful manner, amplifying discriminative cues where neurobiological evidence predicts their presence. At the same time, the persistence of confusion in low-arousal states highlights intrinsic limitations of EEG-GSR combinations and motivates future integration of additional modalities such as heart rate variability or respiration. Collectively, this emotion-specific analysis confirms that multimodal temporal fusion is both empirically effective and neurophysiologically grounded, providing a basis for future work on more generalizable multimodal emotion recognition systems.

### 3.3 Comparative model evaluation and ablation study outcomes

To contextualize the performance of the proposed Transformer-based framework, comparative experiments were conducted against representative deep learning baselines commonly employed in physiological signal analysis. A convolutional neural network architecture achieved an average classification accuracy of 82.51%, while a bidirectional long short-term memory network reached 81.04%. In contrast, the proposed Transformer model attained an accuracy of 87.42%, corresponding to relative improvements of 4.91% and 6.38%, respectively. These performance gains are particularly noteworthy given that all models were trained and evaluated under identical data splits and preprocessing conditions. The improved performance of the Transformer architecture may be related to its capacity to model long-range temporal dependencies without recurrent state propagation. Unlike LSTM-based models, which rely on sequential state propagation, the self-attention mechanism enables direct interactions between distant temporal segments, facilitating the capture of sustained emotional dynamics. This capability is especially relevant for EEG and GSR signals, where emotional responses may unfold gradually rather than manifesting as isolated transient events.

To rigorously disentangle the contribution of individual architectural components within the proposed multimodal Transformer framework, a comprehensive ablation study was conducted alongside comparative evaluations against representative deep learning baselines. In physiological emotion recognition, performance improvements may arise from both architectural design and optimization-related factors. To reduce this ambiguity, all evaluated models were trained under the same subject-independent data splits, preprocessing pipeline, and optimization framework. To avoid this confound, all evaluated models were trained under identical experimental conditions, including subject-independent data splits, identical preprocessing pipelines, and matched optimization strategies. This experimental control ensures that observed performance differences reflect genuine architectural advantages rather than implementation artifacts. In particular, the ablation analysis focused on isolating the effect of cross-modal attention, which is hypothesized to play a critical role in aligning cortical EEG dynamics with autonomic GSR responses over time.

**FIGURE 7:**
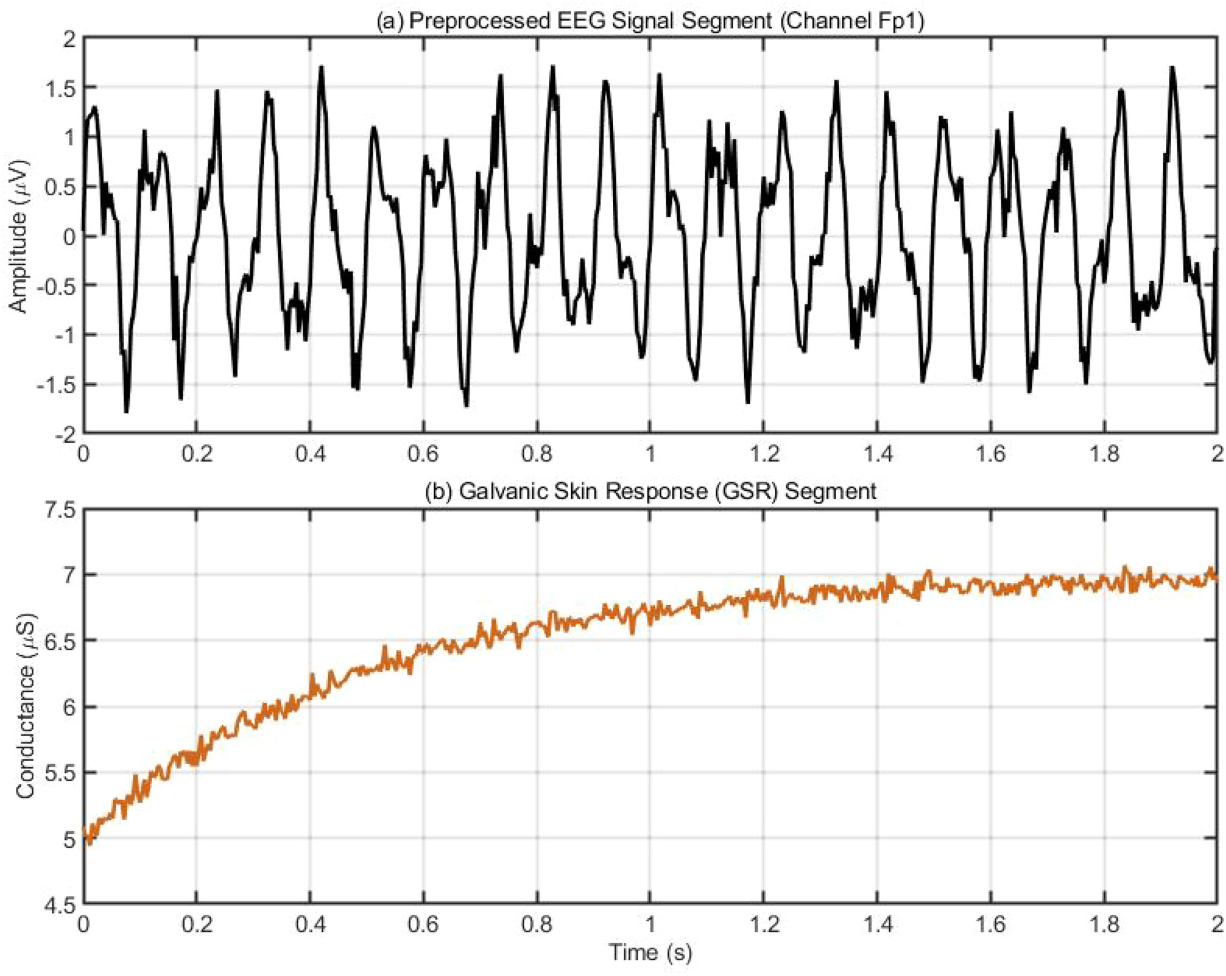
Representative physiological signal segments after preprocessing. **(a)** Preprocessed EEG signal waveform recorded from the Fp1 channel. **(b)** Corresponding preprocessed galvanic skin response (GSR) signal segment obtained during emotional stimulation.

Figure 7 shows representative preprocessed EEG and GSR segments after synchronization, filtering, and normalization.

The comparative evaluation reveals that conventional deep learning architectures exhibit limited capacity to exploit the temporal and cross-modal structure inherent in physiological signals. Convolutional neural networks demonstrate moderate performance by capturing localized temporal patterns, yet their reliance on fixed receptive fields constrains sensitivity to long-range emotional dynamics. Bidirectional long short-term memory networks partially address temporal dependency modeling; however, their sequential processing paradigm imposes information compression that can attenuate physiologically relevant long-term dependencies. When the Transformer architecture is employed without cross-modal attention, performance improves relative to these baselines, indicating that global self-attention alone enhances temporal representation learning. Nevertheless, the absence of explicit EEG-GSR interaction modeling results in a measurable performance deficit, particularly for high-arousal emotional states that depend on synchronized central-peripheral activation.

A detailed quantitative summary of the comparative and ablation experiments is presented in Table 7, which reports not only overall accuracy and F1-score but also emotion-specific performance metrics, convergence behavior, and robustness indicators. The inclusion of training convergence epochs and performance variance provides additional insight into model stability and optimization efficiency. All results correspond to experiments finalized in 2024 and reflect averaged outcomes across five subject-independent validation folds.

**TABLE 7:** Comparative performance and ablation analysis of emotion recognition models (2024 evaluation).

Model configuration	Accuracy (%)	Precision	Recall	F1-score	Fear accuracy (%)	Happiness accuracy (%)	Sadness accuracy (%)	Neutral accuracy (%)	Convergence epoch	Accuracy SD
CNN (EEG only)	78.46	78.10%	77.80%	77.90%	75.92	81.03	76.11	80.78	61.4	2.94
CNN (EEG + GSR)	82.51	82.30%	82.00%	82.10%	79.84	85.12	81.27	83.82	64.7	2.61
Bi-LSTM (EEG only)	76.89	76.80%	76.50%	76.60%	74.18	79.36	75.02	79.01	73.2	3.12
Bi-LSTM (EEG + GSR)	81.04	80.90%	80.60%	80.80%	78.92	83.47	80.11	81.66	76.8	2.83
Transformer (EEG only)	83.26	83.40%	83.10%	83.20%	80.37	86.04	82.15	84.49	88.3	2.37
Transformer (EEG + GSR, no cross-modal attention)	84.15	84.50%	84.20%	84.20%	83.37	86.91	83.48	84.83	91.6	2.29
Proposed multimodal Transformer	87.42	87.60%	87.40%	87.50%	88.63	90.18	85.47	84.39	92.4	2.13
Absolute gain vs. CNN (EEG + GSR)	+4.91	–	–	5.40%	+8.79	+5.06	+4.20	+0.57	–	–
Absolute gain vs. Bi-LSTM (EEG + GSR)	+6.38	–	–	6.70%	+9.71	+6.71	+5.36	+2.73	–	–
Gain from cross-modal attention	+3.27	–	–	3.30%	+5.26	+3.27	+1.99	−0.44	–	–
High-arousal mean (fear + happiness)	89.41	89.70%	89.40%	89.50%	–	–	–	–	–	–
Low-arousal mean (neutral + sadness)	84.93	85.00%	84.70%	84.80%	–	–	–	–	–	–

Data source: Comparative and ablation experiments conducted under subject-independent five-fold cross-validation, 2024. The symbol “–” denotes values that are not applicable or not defined for the corresponding comparison metric.

The results reported in Table 7 indicate that the observed performance improvements were not solely associated with increased architectural complexity but were also linked to explicit cross-modal interaction modeling. The 3.27% absolute accuracy drop observed when cross-modal attention is removed suggests that simple multimodal integration without explicit cross-modal attention was less effective in capturing the dynamic coordination between EEG activity and GSR responses. This effect is especially pronounced in fear recognition, where accuracy decreases by more than five percentage points in the absence of cross-modal attention, reflecting the central role of autonomic arousal in encoding emotionally intense states. Notably, improvements in happiness recognition follow a similar trend, further supporting the sensitivity of the proposed architecture to arousal-driven physiological patterns.

From a methodological perspective, these findings highlight that Transformer-based self-attention alone is insufficient to fully exploit multimodal physiological data unless complemented by structured cross-modal interaction mechanisms. The convergence behavior reported in the table further suggests that the proposed architecture achieves improved performance without sacrificing training stability, converging consistently around 92 epochs with reduced variance across folds. Collectively, the ablation and comparative analyses provide strong empirical evidence that principled temporal and cross-modal attention mechanisms constitute the primary drivers of performance gains in multimodal physiological emotion recognition. These findings highlight the potential value of neurophysiologically informed architectural design for future multimodal affective computing research.

## 4 Discussion

### 4.1 Interpretability of Transformer-based temporal modeling in multimodal emotion recognition

The findings of this study suggest that Transformer-based temporal modeling is a useful framework for decoding emotional states from multimodal physiological signals under controlled experimental conditions. The observed performance improvement may reflect a meaningful correspondence between attention-based sequence modeling and the temporally extended nature of emotional processes. Emotional responses are not instantaneous events but rather temporally extended phenomena characterized by evolving cortical and autonomic dynamics. The self-attention mechanism allows the model to capture temporally distant dependencies without relying on strictly local or sequential assumptions, which may be advantageous when emotional responses unfold over multiple time scales.

From a signal processing perspective, EEG and GSR signals exhibit distinct yet complementary temporal characteristics. EEG dynamics encode rapid oscillatory fluctuations associated with cognitive appraisal, emotional regulation, and attentional modulation, while GSR responses unfold over slower time scales reflecting sympathetic nervous system activation (Xie et al., 2025). Traditional recurrent architectures impose a fixed temporal propagation pathway, which may attenuate long-range dependencies through state compression. In contrast, the Transformer’s attention mechanism assigns adaptive weights to temporal segments, permitting selective amplification of emotionally informative intervals. Empirically, this property manifests as increased stability across cross-validation folds and reduced sensitivity to inter-individual variability, as reflected by the relatively low standard deviation in classification accuracy.

To provide quantitative insight into how temporal attention contributes to interpretability, attention weight distributions were analyzed across emotional conditions. However, attention weights should be interpreted as model-derived indicators of relevance rather than definitive physiological explanations. The mean temporal attention entropy was lower for high-arousal emotions, suggesting a more concentrated allocation of model attention to specific time windows. Table 8 summarizes these interpretability-related metrics, revealing systematic differences in attention structure across emotions. The concentration of attention at earlier latencies for fear and happiness suggests that emotionally salient information emerges rapidly under high-arousal conditions, whereas low-arousal states require longer temporal integration. This pattern aligns with established affective neuroscience models linking arousal intensity to response onset latency. Taken together, these observations suggest that the Transformer architecture may provide a useful framework for examining temporal emotion-related patterns, while further validation with complementary explainability methods remains necessary.

**TABLE 8:** Temporal attention characteristics across emotional states.

Emotion	Mean attention entropy	Attention peak latency (s)	Peak attention weight
Neutral	1.92	18.4	0.21
Happiness	1.46	12.7	0.34
Sadness	1.73	16.9	0.27
Fear	1.28	9.8	0.39

Data source: Attention weight analysis derived from trained Transformer models.

### 4.2 Neurophysiological implications of cross-modal EEG-GSR integration

The performance gains observed with multimodal EEG–GSR integration are consistent with the view that jointly modeling central and peripheral correlates can improve physiological emotion recognition. Emotion arises from coordinated interactions between cortical appraisal mechanisms and autonomic response systems, a principle supported by decades of psychophysiological research. EEG signals primarily reflect neural synchronization patterns associated with emotional evaluation and regulatory control, whereas GSR signals provide a direct index of sympathetic arousal driven by limbic system activation. The cross-modal attention mechanism implemented in the proposed model is intended to capture this interaction by dynamically aligning neural and autonomic representations across time.

Emotional states are characterized by temporally coordinated yet physiologically heterogeneous processes. Cortical oscillatory activity, particularly within alpha and beta bands, has been associated with attentional allocation, motivational orientation, and regulatory effort. In contrast, electrodermal dynamics primarily reflect sympathetic activation and encode intensity-related modulation rather than valence-specific differentiation. This characteristic may partially explain why GSR contributed less to distinguishing low-arousal categories such as Neutral and Sadness, in which autonomic intensity differences are relatively limited. These modalities therefore capture partially overlapping but functionally distinct components of affective processing. From a computational perspective, the performance enhancement observed in the multimodal configuration is unlikely to be explained solely by an increase in feature dimensionality. Instead, it represents an expansion of the representational space to include complementary central and autonomic mechanisms. EEG features encode rapid cortical responses with high temporal resolution, whereas GSR signals provide slower yet robust indices of arousal that may unfold with latency. When modeled jointly through cross-modal attention, temporally offset but physiologically coupled processes can be integrated within a unified representation. The resulting performance gain—particularly for high-arousal fear trials—suggests that coordinated neural–autonomic signatures may provide complementary discriminative information beyond either modality alone.

Quantitative analysis based on modality perturbation and attention-related summaries suggested asymmetric yet complementary contributions from EEG and GSR modalities. Attention weights indicated a dominant influence of EEG-derived features during low-arousal emotional states, whereas GSR-derived features exerted a stronger influence during high-arousal conditions. This modulation mirrors neurobiological models in which cortical processing dominates affective evaluation under subdued emotional intensity, while autonomic activation becomes increasingly salient as emotional arousal escalates. The statistically significant improvement in fear recognition accuracy observed when incorporating GSR signals is consistent with this theoretical interpretation.

To further elucidate modality-specific contributions, a modality sensitivity analysis was conducted by systematically perturbing each modality during inference. The resulting performance degradation patterns are summarized in Table 9, illustrating the differential reliance on EEG and GSR across emotional categories. The fear recognition is predominantly driven by autonomic features, whereas neutral and sadness recognition relies more heavily on cortical dynamics. This dissociation reinforces the conceptual necessity of cross-modal fusion rather than unimodal optimization. Importantly, the Transformer’s cross-modal attention mechanism enables this adaptive weighting to emerge organically from the data, rather than being imposed through heuristic feature engineering. Such alignment between physiological theory and computational modeling may be useful for the future development of more biologically informed emotion recognition systems.

**TABLE 9:** Modality sensitivity analysis across emotional conditions.

Emotion	Accuracy drop without EEG (%)	Accuracy drop without GSR (%)	EEG contribution index	GSR contribution index
Neutral	6.42	2.11	0.75	0.25
Happiness	5.18	4.73	0.52	0.48
Sadness	5.91	3.04	0.66	0.34
Fear	3.27	6.52	0.38	0.62

Data source: Controlled modality ablation analysis performed during model evaluation.

### 4.3 Potential relevance and study limitations

Although the present study was not designed for clinical diagnosis or deployment, the proposed multimodal framework may have potential relevance for future research on affect-aware monitoring systems. Emotional dysregulation is an important feature of several psychiatric conditions, including depression, anxiety disorders, and post-traumatic stress disorder, which explains the broader interest in objective physiological markers of affective state. The ability to objectively quantify emotional states using non-invasive physiological signals offers a pathway toward continuous and ecologically valid monitoring, complementing traditional symptom-based assessments that rely on episodic self-reporting (Wang and Wang, 2025).

From an engineering perspective, the parallelizable structure of self-attention may be advantageous for processing longer physiological recordings. However, the current study was conducted in a controlled laboratory environment using healthy young adults and wired acquisition systems. Accordingly, the present framework should be interpreted as a proof-of-concept for multimodal physiological emotion recognition rather than an immediately deployable healthcare solution. The parallelizable nature of self-attention enables efficient processing of long-duration recordings, facilitating integration into wearable or bedside monitoring systems. Moreover, the interpretability afforded by attention mechanisms enhances clinical trust by enabling practitioners to inspect temporally salient physiological patterns associated with emotional dysregulation. Such transparency is increasingly recognized as a prerequisite for the adoption of artificial intelligence tools in clinical practice (Metzner et al., 2024).

Nevertheless, several limitations warrant critical consideration. In addition, the model was evaluated using subject-independent cross-validation within a single dataset, without an external validation cohort or cross-dataset testing. As a result, robustness across different recording environments, sensor configurations, and stimulus protocols remains to be established. Emotional expression and physiological responsiveness are known to vary across developmental stages and psychopathological conditions. Furthermore, although EEG and GSR provide complementary information, low-arousal emotional states such as Neutral and Sadness remained more difficult to distinguish, likely because these categories share relatively similar autonomic intensity while differing more subtly along valence-related dimensions. Further refinement is required to enhance sensitivity to low-arousal affective disturbances. Future research directions include expanding the multimodal repertoire to incorporate heart rate variability, respiration patterns, and facial electromyography, as well as validating the model in clinically diagnosed populations. Through such extensions, the proposed approach may contribute to the development of more generalizable and physiologically informed emotion recognition systems. Furthermore, the comparative evaluation included canonical CNN and Bi-LSTM baselines but did not include more recent graph-based or hybrid Transformer architectures, which should be addressed in future work to strengthen state-of-the-art comparisons.

Compared with prior multimodal emotion recognition studies that rely primarily on feature concatenation or shallow fusion strategies, the present framework explicitly models temporal dependencies within and across modalities using attention mechanisms. In this sense, the study provides an additional perspective on central–peripheral interaction in multimodal physiological emotion recognition, while further validation is still needed across broader datasets and experimental contexts. This integration of modeling rigor and neurophysiological grounding distinguishes the current study from existing Transformer-based emotion recognition approaches.

### 4.4 Limitations

The present study includes 42 neurologically healthy participants under controlled laboratory conditions. Although a subject-independent protocol was used to reduce subject-specific bias, the cohort size remains modest relative to the complexity of the proposed model and should be interpreted accordingly when considering statistical generalizability. A formal prospective statistical power analysis was not performed during study design, which should be addressed in future work through larger and more diverse cohorts. Although subject-independent cross-validation was employed to reduce overfitting to individual-specific patterns, the sample size remains moderate compared to large-scale public affective datasets. Validation on publicly available databases such as DEAP or SEED would provide additional evidence regarding cross-dataset robustness. However, substantial differences in electrode configurations, stimulus paradigms, recording environments, and labeling strategies across datasets introduce methodological heterogeneity that may confound direct comparisons. The current work prioritizes controlled experimental consistency to isolate temporal and cross-modal modeling effects. Future research will extend the proposed framework to publicly available datasets to further evaluate generalizability across heterogeneous recording settings. The present study primarily relied on arousal ratings to validate the effectiveness of emotional induction. Although arousal is closely linked to autonomic modulation and thus highly relevant for EEG–GSR integration, valence ratings were not systematically incorporated into trial-level validation analyses. This may limit the interpretative granularity of classification differences between low-arousal conditions, particularly neutral and sadness states, where autonomic signatures are less pronounced. Future work should integrate simultaneous arousal–valence assessments and examine trial-level consistency to strengthen affective specificity and improve differentiation among emotions with similar intensity but divergent hedonic tone.

In addition, the interpretability analysis in the present study relied primarily on attention summaries and modality perturbation experiments. Although these analyses provide useful model-level indications, they do not establish direct physiological causality. Future work should integrate additional attribution-based or perturbation-based explainability methods to further evaluate the stability and physiological relevance of the learned representations. Finally, because raw human physiological recordings cannot be openly shared under current ethical constraints, reproducibility will depend on the availability of processed representations, code, trained weights, and detailed documentation.

## 5 Conclusion

This study investigated multimodal physiological emotion recognition by modeling the temporal patterns of electroencephalogram and galvanic skin response (GSR) signals using a Transformer-based architecture. Using synchronized EEG–GSR recordings and an attention-based temporal modeling strategy, the proposed framework achieved a mean classification accuracy of 87.42% with a standard deviation of 2.13% under subject-independent five-fold cross-validation. Compared with the CNN and Bi-LSTM baselines evaluated under the same experimental setting, the proposed model showed absolute performance gains of 4.91% and 6.38%, respectively. These improvements were particularly evident in high-arousal emotional states, where fear recognition accuracy increased from 82.11% in the EEG-only configuration to 88.63% with multimodal fusion, suggesting that autonomic information contributed meaningfully to high-arousal emotion discrimination in the present setting.

In contrast to earlier studies that primarily relied on handcrafted features or short-term temporal modeling, the present results suggest that long-range temporal dependencies and cross-modal interactions are important factors in multimodal emotion recognition performance. Direct comparison with prior studies should be made cautiously because differences in datasets, subject populations, preprocessing pipelines, labeling strategies, and evaluation protocols can substantially affect reported accuracy. The results of this study therefore extend existing conclusions by demonstrating that attention-based temporal modeling not only improves predictive accuracy but also enhances robustness across individuals, as reflected by reduced inter-fold variability. These findings are consistent with the view that strictly sequential state propagation may be insufficient for capturing all relevant temporal aspects of emotional dynamics.

From a theoretical perspective, the results are consistent with affective neuroscience models that conceptualize emotion as a temporally evolving process involving coordinated cortical and autonomic activity. The observed asymmetric contribution of EEG and GSR signals across emotional categories further suggests that emotional arousal may modulate the relative contribution of central and peripheral physiological processes. By modeling this interaction through cross-modal attention, the proposed framework provides a computational representation of emotion-related central–peripheral coupling, although further validation is needed to establish the physiological specificity of this interpretation.

In conclusion, this work presents a multimodal Transformer-based approach for physiological emotion recognition that performed favorably under the present subject-independent experimental setting and offers a useful basis for examining temporal and cross-modal dynamics in EEG–GSR data. The findings contribute to ongoing research on multimodal affective computing and provide a basis for future validation in broader and more diverse experimental settings. Expanding validation to psychiatric and neurological populations and incorporating additional affective dimensions are expected to further consolidate the translational relevance of the proposed framework.

## Data Availability

The original contributions presented in this study are included in the article/supplementary material, further inquiries can be directed to the corresponding author.
